# Identification of Putative Receptors for the Novel Adipokine CTRP3 Using Ligand-Receptor Capture Technology

**DOI:** 10.1371/journal.pone.0164593

**Published:** 2016-10-11

**Authors:** Ying Li, Tammy Ozment, Gary L. Wright, Jonathan M. Peterson

**Affiliations:** 1 Quillen College of Medicine, Department of Biomedical Sciences, East Tennessee State University, Johnson City, Tennessee, United States of America; 2 Quillen College of Medicine, Department of Internal Medicine, East Tennessee State University, Johnson City, Tennessee, United States of America; 3 College of Public Health, Department of Health Sciences, East Tennessee State University, Johnson City, Tennessee, United States of America; Thomas Jefferson University, UNITED STATES

## Abstract

C1q TNF Related Protein 3 (CTRP3) is a member of a family of secreted proteins that exert a multitude of biological effects. Our initial work identified CTRP3’s promise as an effective treatment for Nonalcoholic fatty liver disease (NAFLD). Specifically, we demonstrated that mice fed a high fat diet failed to develop NAFLD when treated with CTRP3. The purpose of this current project is to identify putative receptors which mediate the hepatic actions of CTRP3.

**Methods:**

We used Ligand-receptor glycocapture technology with TriCEPS™-based ligand-receptor capture (LRC-TriCEPS; Dualsystems Biotech AG). The LRC-TriCEPS experiment with CTRP3-FLAG protein as ligand and insulin as a control ligand was performed on the H4IIE rat hepatoma cell line.

**Results:**

Initial analysis demonstrated efficient coupling of TriCEPS to CTRP3. Further, flow cytometry analysis (FACS) demonstrated successful oxidation and crosslinking of CTRP3-TriCEPS and Insulin-TriCEPS complexes to cell surface glycans. Demonstrating the utility of TriCEPS under these conditions, the insulin receptor was identified in the control dataset. In the CTRP3 treated cells a total enrichment of 261 peptides was observed. From these experiments 5 putative receptors for CTRP3 were identified with two reaching statistically significance: Lysosomal-associated membrane protein 1 (LAMP-1) and Lysosome membrane protein 2 (LIMP II). Follow-up Co-immunoprecipitation analysis confirmed the association between LAMP1 and CTRP3 and further testing using a polyclonal antibody to block potential binding sites of LAMP1 prevented CTRP3 binding to the cells.

**Conclusion:**

The LRC-TriCEPS methodology was successful in identifying potential novel receptors for CTRP3.

**Relevance:**

The identification of the receptors for CTRP3 are important prerequisites for the development of small molecule drug candidates, of which none currently exist, for the treatment NAFLD.

## Introduction

Since the discovery of leptin by Zhang et al. [1] many secreted bioactive molecules have been identified which originate from adipose tissue. Thus far, over 260 unique adipose tissue derived secreted proteins/peptides have been identified, collectively termed adipokines [2–5]. Efforts to identify such metabolic regulators have led to the discovery of a family of secreted proteins, designated as C1q TNF-Related Proteins, with 15 unique proteins currently identified (CTRP1-15) [6–14]. C1q family proteins are characterized by a distinctive ‘globular domain’ of about 140 amino acids (the gC1q domain) [14]. The CTRP proteins, adiponectin, TNF-alpha, as well as other proteins with the C1q domain are collectively referred to as the C1q/TNF superfamily [14–18]. Proteins within the C1q/TNF superfamily share some structural similarities, but may have apposing functions [18]. To date, many unique functions have been identified for the CTRP proteins encompassing regulatory roles in metabolism, inflammation and cell proliferation [6, 9, 15–29]. Of these proteins our lab has identified a liver specific role for CTRP3 in preventing Nonalcoholic fatty liver disease (NAFLD) [29].

Adiponectin, the most widely studied member of the C1q/TNF superfamily, increases lipid oxidation in liver and skeletal muscle [16, 30–32]. Unlike adiponectin or other C1q TNF related proteins, we observed no direct effect of CTRP3 on skeletal muscle *in vitro* or *in vivo [9, 29]*. This implies that CTRP3 works through a novel receptor, as the three identified receptors for adiponectin are all present in skeletal muscle [33–35]. In further support of this hypothesis both CTRP3 and adiponectin decrease hepatic TAG accumulation, however adiponectin reduces hepatic triglyceride levels largely through activation of AMP-activated protein kinase (AMPK) pathways [32], whereas CTRP3 did not affect AMPK phosphorylation status [9, 29]. The finding of divergent downstream signaling pathways also argue that CTRP3 has a receptor distinct from adiponectin. Combined, these data indicate that CTRP3 is a distinct member of the C1q/TNF superfamily and functions through a unique receptor in liver. The receptor and the mechanism(s) responsible for the CTRP3-induced reduction in hepatic TAG accumulation remain unexplored.

LRC-TriCEPS™ is an promising, novel technology recently described in Nature Protocols and Nature Biotechnology [36, 37]. First published by the lab of Bernd Wollscheid, TriCEPS™ is a chemoproteomic reagent coupled to a ligand of interest (CTRP3) that acts to covalently link the ligand to the cell-based receptor [36]. This linkage protects the ligand-receptor conjugate from the subsequent digestion steps and peptide level purification via the biotin tag on the TriCEPS™ molecule. The TriCEPS™ and the ligand are subsequently released through specific enzymatic cleavage and the receptor peptides are collected and analyzed by Liquid chromatography-tandem mass spectrometry (LC-MS/MS). The LRC-TriCEPS™ technique has been validated through successful detection of known receptors, however, using this technology to identify receptors for novel proteins is still in its infancy [36, 37].

The purpose of this study was to determine if we could successful use the LRC-TriCEPS™ to identify novel potential receptors which mediate the hepatic effects of CTRP3. In brief, we were able to identify five potential novel receptors using the LRC-TriCEPS™ technique.

## Methods

### Cell culture

HEK-293T cells (Thermo fisher, GripTite 293 MSR Cell Line Cat # R79507) were cultured in Dulbecco's Modification of Eagle's Medium (DMEM) with 4.5 g/L glucose without L-glutamine and sodium pyruvate (Corning, Cat# 15–017) supplemented with 5% (v/v) fetal bovine serum (HyClone^TM^, Cat# SH30088.03) and with antibiotic/antimycotic Solution (Corning, Cat# 30-004-CI). HEK-293T cells were used for transfection and protein purification protocols. H4IIE cells (H-4-II-E rat hepatoma cells, ATCC® cat# CRL-1548™, RRID:CVCL_0284) were cultured in DMEM containing 1 g/L glucose, L-glutamine, and sodium pyruvate (Corning Cat# 10–014) supplemented with 10% (v/v) fetal bovine serum (HyClone^TM^, cat# SH30088.03) and with antibiotic/antimycotic Solution (Corning, Cat# 30-004-CI). H4IIE rat hepatoma cells are a well-established *in vitro* model of hepatocytes, useful for metabolic research as this cell line mirrors the liver-like, insulin regulated glucose and lipid metabolism found in the liver [38–40]. Further, the CTRP3 amino acid sequence is highly conserved throughout vertebrate evolution with only 4 amino acids differing between the mouse and rat orthologs and a 95% homogeneity between mouse and human [6, 9]. Therefore, we expect that the receptor and metabolic effects of CTRP3 established in H4IIE cell line *in vitro* will provide insight to the actions of CTRP3 *in vivo*.

### Protein purification

C-terminal FLAG-Tagged mouse CTRP3 and CTRP1 were produced as described previously [9, 41]. Briefly, transient transfections were performed on HEK-293T cells using calcium phosphate according to standard protocols [42]. At 48 h after transfection, cells were washed and then cultured in serum-free Opti-MEM I medium (Invitrogen, Cat# 51985034) supplemented with vitamin C (0.1 mg/ml; Fisher Scientific Company, Cat# FLBP351). Supernatants were collected three times, every 48 h, pooled and purified using an anti-FLAG affinity gel according to the manufacturer's protocol (Biotool.com, Cat# B23101), and eluted with 150 μg/ml FLAG peptide (Sigma, Cat# F4799). Dialysis was performed on purified proteins with 20 mM Hepes buffer (pH 8.0) containing 135 mM NaCl in a 10 kDa cut-off Slide-A-Lyzer dialysis cassette (Thermo Fisher Scientific, Cat# 88252).

### Immunofluorescence

For visualization H4IIE hepatocytes were grown to confluence on Millicell EZ SLIDE (Millipore Cat# PEZGS0816) in standard growth medium. The cells treated with recombinant FLAG tagged CTRP3, CTRP1, or vehicle for 1 hr. Afterwards the cells were washed in phosphate buffered saline (PBS; 137 mM NaCl, 10 mM Phosphate, 2.7 mM KCl, ph 7.2) fixed in 4% formaldehyde diluted in PBS for 10 minutes at 37 C, washed with PBS, and then blocked in 5% Normal goat serum (diluted in PBS). The cells were then incubated with rabbit anti-FLAG (Cell Signaling Technology Cat# 14793; RRID: AB_2572291) followed by fluorochrome-conjugated secondary antibody (Cell Signaling Technology Cat# 4412 RRID: AB_1904025). Cells were then mounted with an anti-fade mounting medium with DAPI (DAPI Fluoromount-G®; SouthernBiotech Cat# 0100–20), and immunofluorescence was visualized [Zeiss Observer.Z1].

### Fatty acid oxidation

H4IIE hepatocytes were allowed to adhere to 24-well culture plates XF24 cell culture microplate (Seahorse Bioscience, Cat# 100777–004) after seeding for 2 days, according to standard protocols (Seahorse Bioscience). Cells were pre-incubated with 5 μg/ml CTRP3 or vehicle for 1 H then transferred into XF assay medium (Seahorse Bioscience, Cat# 100965–000) supplemented with 0.5mM sodium pyruvate and 5mM glucose before being placed into the XF24 Extracellular Flux Analyzer (Seahorse Bioscience XF24). The dosage of recombinant CTRP3 was selected based on our previous experimental observations [9], and is well within the common dosages reported within the literature of 2–30 μg/ml [9, 29, 43, 44]. Sensor cartridge of XF24 extracellular flux assay kit (Seahorse Bioscience, Cat# 100850–001) was hydrated by loading 1ml XF calibrant (Seahorse Bioscience, Cat# 100840–000) into each well in utility plate and incubating at 37°C overnight in a non-CO_2_ incubator. 200uM palmitate conjugated to bovine serum albumin (BSA) or fatty acid free BSA (vehicle control) was added in XF24 cell culture microplate at time-points indicated. A 1 mM working stock of palmitate (Sigma Cat# P5585) conjugated to 0.17 mM fatty acid free BSA (Sigma Cat# A8806) was prepared according to established protocols (Seahorse Bioscience). Briefly, 50 ml of a BSA solution (0.34 mM BSA, 150 mM NaCl, pH 7.4) was heated to 37 C and 40 ml of a palmitate solution (2.98 mM palmitate, 150 mM NaCL), heated to 70 C, and was added in 5 mL increments. The combined solution was incubated at 37 C for 1 H under constant agitation, afterwards the pH was adjusted to 7.4 and final volume was adjusted to 100 mL with 150 mM NaCl). Aliquots were stored until use at -20 C in glass vials.

### Flow Cytometry

H4IIE hepatocytes were grown to confluence in 6-well plates (Corning Costar® Cat# 3516) and then treated with/without recombinant CTRP3-FLAG as indicated.The cells were then collected in PBS, fixed in 4% formaldehyde, and then incubated with rabbit anti-FLAG antibody (Cell Signaling Technology, Cat# 14793; RRID: AB_2572291) followed by fluorochrome-conjugated secondary antibody (Cell Signaling Technology, Cat# 4412; RRID:AB_1904025). Next cells were suspended in buffer (0.5% Bovine Serum Albumin in PBS) and analyzed for mean fluorescent intensity (MFI) by using a FACScalibur flow cytometer with (CellQuest software, BD Biosciences). Except for the quality control step for LRC-TriCEPS (see below), all FACS experiments were performed three separate times in triplicate for each experiment (S1–S3 Files). For blocking experiments cells were co-incubated with polyclonal Lysosomal-associated membrane protein 1 (LAMP-1) antibody (Santa Cruz Biotechnology Cat# sc-8098, RRID:AB_2134494)

### TriCEPS™-based ligand-receptor capture (LRC-TriCEPS)

In conjunction with Dualsystems Biotech AG, TriCEPS™-based ligand-receptor capture (LRC-TriCEPS; Dualsystems Biotech, cat# P05201) was utilized to identify the putative receptor for CTRP3 according to manufacturer’s directions. Briefly, 300μg recombinant CTRP3-FLAG protein or Insulin (control ligand) was dissolved in 150 μl HEPES buffer (25 mM, pH 8.2) and 1.5 μl of the TriCEPS™ reagent was added to each sample and incubated for 2 H at 20°C under constant agitation. After incubation 1μl of each sample was removed to complete a Dot blot experiment as a quality control to test for efficient TriCEPS™ coupling to the ligands (data not shown). Three separate 50 mL tubes each containing 2x10^8^ H4IIE Hepatocytes in PBS (pH 6.5) were washed and cooled to 4°C, all subsequent steps were performed at 4°C. 200 μl was collected from each tube and labeled as non-oxidized cells. Next, to mildly oxidize the cell surface proteins 1.5 mM NaIO_4_ was added to each tube and cells were incubated at 4°C in the dark for 15 min under gentle rotation. The cells were then washed twice at 300 x g for 5 min and then resuspended in 20 ml PBS (pH 6.5). An aliquot of ~80 μl was collected from each tube and labeled as oxidized cells. The cells were then evenly divided into 6 separate tubes and 50 μl of either TriCEPS™-coupled insulin or TriCEPS™-coupled CTRP3 was added to each tube and incubated for 90 min at 4°C under constant gentle agitation. An aliquot of ~80 μl was collected from each tube and the collected samples (non-oxidized cells, oxidized cells, insulin cells and CTRP3-FLAG cells) were analyzed by FACS to test for the crosslinking efficiency of the TriCEPS™-ligand complexes to the cell surface glycans using fluorochrome conjugated streptavidin (Thermo Fisher Scientific Inc. eBioscience Cat# 11–4317). After completion of the coupling reaction, the cells were collected and the cell pellet was sent to Dualsystems for LC-MS/MS analysis.

### LC-MS/MS analysis

The samples were analyzed on a Thermo LTQ Orbitrap XL spectrometer fitted with an electrospray ion source. The samples were measured in data dependent acquisition mode in a 60 min gradient using a 10cm C18. The six individual samples in the dataset were analyzed with a statistical ANOVA model. This model assumes that the measurement error follows Gaussian distribution and views individual features as replicates of a protein's abundance and explicitly accounts for this redundancy. It tests each protein for differential abundance in all pairwise comparisons of ligand and control samples and reports the p-values. Next, p-values are adjusted for multiple comparisons to control the experiment-wide false discovery rate (FDR). The adjusted p-value obtained for every protein is plotted against the magnitude of the fold enrichment between the two experimental conditions. Proteins are considered significant if FC≥2 and adj. p-value<0.05.

### Co-Immunoprecipitation (Co-IP) and Immunoblot Analysis

H4IIE hepatocytes were grown to confluence in 6-well plates (Corning Costar® Cat# 3516) and then treated overnight with/without recombinant CTRP3-FLAG overnight. The cells were then washed with PBS and collected in Non-denaturing lysis buffer (20 mM Tris HCl pH 8, 137 mM NaCl, 10% glycerol, 1% Nonidet P-40, 2 mM EDTA). Cells were incubated with constant agitation for 30 minutes at 4° and then centrifuged at 10,000xg at 4°C for 10 minutes and the supernatant was collected and protein concentration was determined via Bradford assay (Pierce Coomassie Plus Cat# 23238). Equal amounts of protein were dilute to 500 μl final volume and used for immunoprecipitation according to manufactures directions (Bimake, Anti-DYKDDDDK(Flag) Affinity Gel Cat# B23101). Total protein homogenate or immunoprecipitate were loaded and separated on a 12% Mini-Protean® TGX^TM^ gel (Bio-Rad, Cat#456–1046) and transferred to nitrocellulose membranes (0.45 μm, Bio-Rad Cat#1620115). Membranes were blocked in 2% non-fat milk and probed with primary antibodies overnight at 4°C (Anti-LAMP1, Abcam Cat# ab24170) followed by horseradish peroxidase (HRP)-conjugated goat anti-rabbit secondary antibody (Thermo Fisher Scientific Cat# 31460, RRID:AB_228341). Membranes were incubated with HRP Substrate (Millipore Immobilon Cat# WBKLS0100) and chemiluminescence was then visualized with FluorChem® M imager (ProteinSimple). Precision Plus Protein™ Dual Color Standards molecular weight markers were used in all immunoblot analysis (BioRad Cat#161–0374).

### Statistical Analysis

For analysis of flow cytometry and fatty acid oxidation, data from each experiment was normalized to the control and the combined data from the three independent replicates was combined and analyzed with a one-way ANOVA (flow cytometry) or repeated measure ANOVA (fatty acid oxidation) followed by Tukey's multiple comparisons test Post hoc analysis. Statistics analysis were completed by Graphpad Prizm version 6.

## Results

### CTRP3 binds and has a physiological effect on hepatoma cells

**Fig 1:**
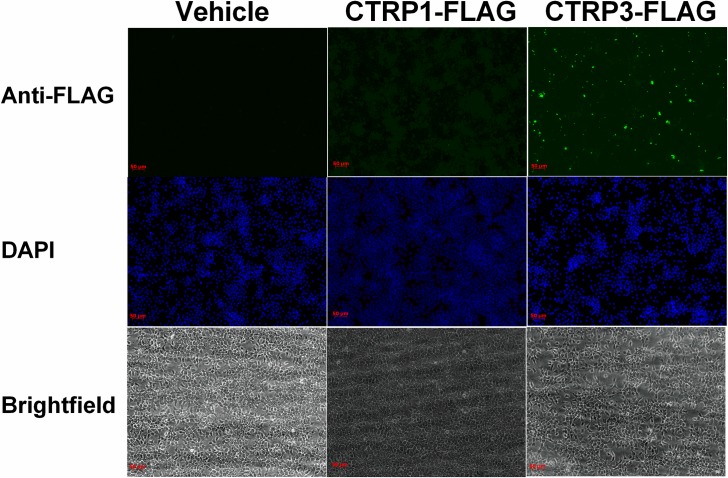
CTRP3 binds to hepatocytes *in vitro*. H4IIE hepatocytes were plated on Millicell EZ SLIDE (Millipore) and allowed to adhere for 48 H in standard growth medium. The cells were treated with recombinant FLAG tagged CTRP3, CTRP1, or vehicle the cells were then incubated with rabbit anti-FLAG primary antibody followed by fluorochrome-conjugated secondary antibody. Cells were then mounted with an anti-fade mounting medium with DAPI, and immunofluorescence was visualized.

**Fig 2:**
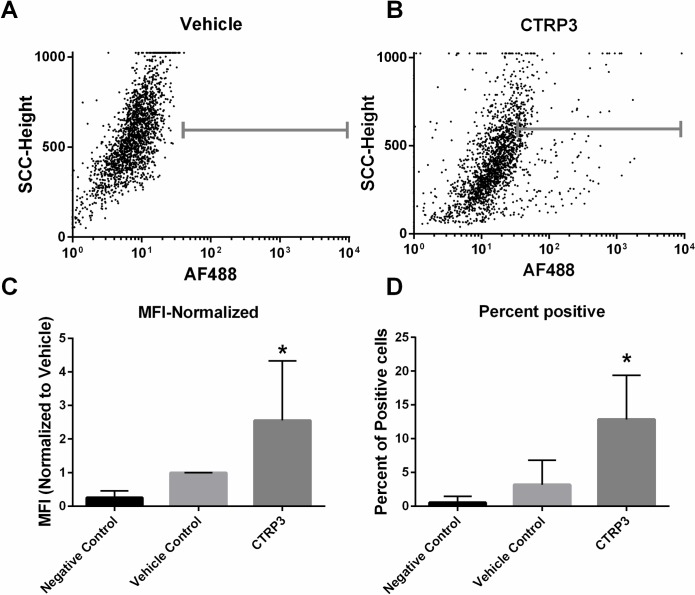
CTRP3 binds to hepatocytes *in vitro*. H4IIE hepatocytes were grown in standard media and then treated for 1 H ± CTRP3-FLAG (5 μg/ml). The cells were then washed and collected in PBS, fixed in 4% formaldehyde, and then incubated with rabbit anti-FLAG antibody followed by fluorochrome-conjugated secondary antibody and analyzed for mean fluorescent intensity (MFI) by flow cytometry. Representative image (20% of points shown) of raw flow data from vehicle treated (A) or CTRP3-FLAG treated (B). C, the MFI ± CTRP3 normalized to vehicle for each independent replicate. D, Percent of the cells that were positive from each experiment. Data for C & D are from 3 independent replicates performed on different days with separate lots of recombinant CTRP3-FLAG protein with each replicate performed in triplicate and reported as mean ± SD. Raw flow cytometry files are attached as supplementary data (S1 File). * p < vs. 0.0001 vehicle.

Immunohistochemical analysis was used to demonstrate that recombinant CTRP3, but not CTRP1 binds to H4IIE hepatocytes *in vitro* (Fig 1). Confirmatory experiments using flow cytometry demonstrated a 200% increase in mean fluorescent intensity (MFI) when cells were treated with recombinant FLAG tagged CTRP3 and probed with anti-FLAG antibodies, compared with vehicle, and fluorochrome-conjugated secondary antibody alone treatments (Fig 2C). Interestingly, only a subset (~20%) of H4IIE cells stained positive for CTRP3 binding. This may indicate that CTRP3 receptor surface expression is transient. For instance, it may be yoked to the cell cycle or some other parameter of cellular circumstance.

**Fig 3:**
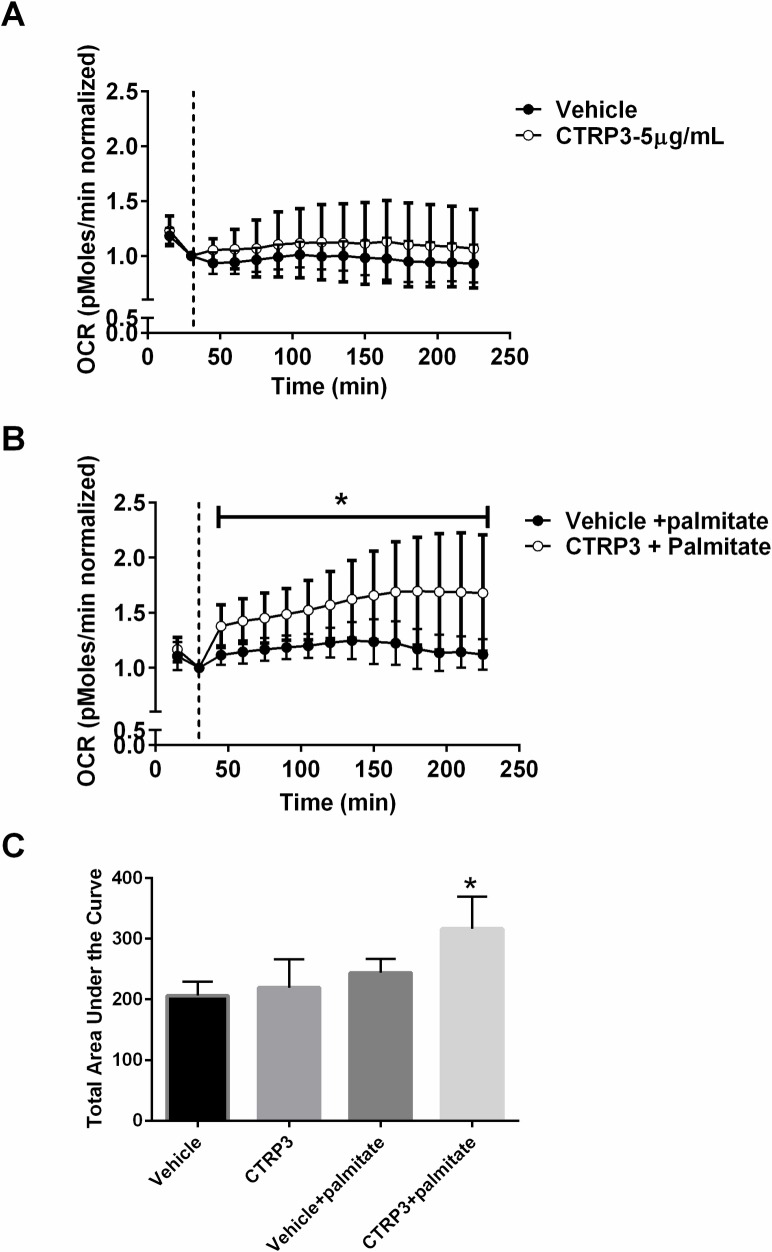
CTRP3 increases oxygen consumption. Cells were pre-incubated with 5 μg/ml recombinant CTRP3 or vehicle for 1 H before being placed into the XFe24 Extracellular Flux Analyzer (Seahorse Bioscience) and oxygen consumption rate (OCR) was determined. OCR was measured in the absence (A) or after the addition of 200 μM palmitate (B) added at 15 minutes (vertical line). C, Area under the curve was calculated (mean OCR value at each interval*time) for each treatment. Data represents the mean ± SD. Data represents pooled data from 3 independent experiments each performed in triplicate, *p < 0.05 vs. vehicle + palmitate.

The next series of experiments examined the effects of CTRP3 on hepatocyte oxygen consumption to determine if the binding of CTRP3 to the hepatocytes resulted in a physiological effect. Pre-treatment with recombinant CTRP3 (5 μg/ml) had no effect on lipid oxidation in hepatocytes under standard conditions. However, in the presence of an excess of free fatty acids (200 μM palmitate) there was an 24% increase in total oxygen consumption in the CTRP3 pre-treated cells, indicating greater FFA utilization (Fig 3).

### TriCEPS™-based ligand-receptor capture (LRC-TriCEPS)

**Fig 4:**
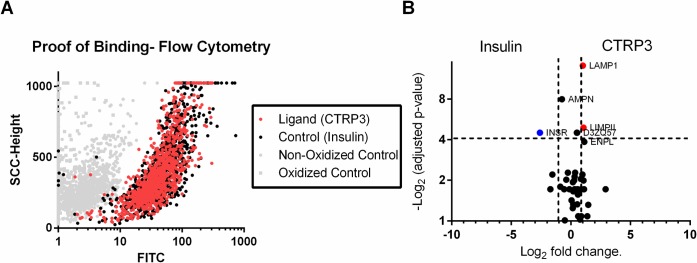
H4IIE rat hepatoma cells were treated with TriCEPs conjugated to Insulin or CTRP3. A, The binding of ligands to cell surface receptors was detected by Steptavidin FITC (The TriCEPS™ reagent contains biotin) and analyzed for mean fluorescent intensity (MFI). Representative image (20% of points shown) of raw flow data from vehicle treated. B, The samples were analyzed by Mass spectrometry (Dualsystems Biotech, AG) and the adjusted p-value (ANOVA, adjusted for multiple comparisons) for the differential abundance of each protein was plotted against the magnitude of the fold enrichment between insulin and CTRP3 samples. Proteins are considered significant if fold change >2 and p<0.05. Two proteins (LAMP1 and LIMPII) were statistically significant. Raw data for Fig 4, S2 File.

An experiment with CTRP3-FLAG protein as a ligand and insulin as a control ligand was performed on the H4IIE rat hepatoma cell line. Control experiments to assess the technical quality were performed in parallel. Briefly, flow cytometry showed successful oxidation and crosslinking of CTRP3-TriCEPS and Insulin-Triceps to the cell surface glycans (Fig 4A). LC-MS/MS analysis showed a total enrichment of glycopeptides of 11% (261 peptides). Under these conditions, INSR (insulin receptor) could be identified and quantified in the control dataset. From these experiments 5 putative receptors for CTRP3 were identified with two reaching statistically significance: Lysosomal-associated membrane protein 1 (LAMP-1) and Lysosome membrane protein 2 (LIMP II) (Table 1 and Fig 4B). As LAMP1 was the promising candidate further experiments were carried out focusing on LAMP1.

**Table 1:** TriCEPS™-based ligand-receptor capture (LRC-TriCEPS).

*Uniprot identifier*	*Protein*	*Number of peptides*	*log2FC*	*adj.pvalue*
*P14562*	LAMP1	4	1.00	5.79E-05
*P27615*	LIMPII	2	1.06	0.035
*P16391*	HA12	3	0.53	0.046
*D3ZQ57*	D3ZQ57	2	0.50	0.046
*Q66HD0*	ENPL	2	1.13	0.072

### LAMP1 antibody prevents CTRP3 binding

**Fig 5:**
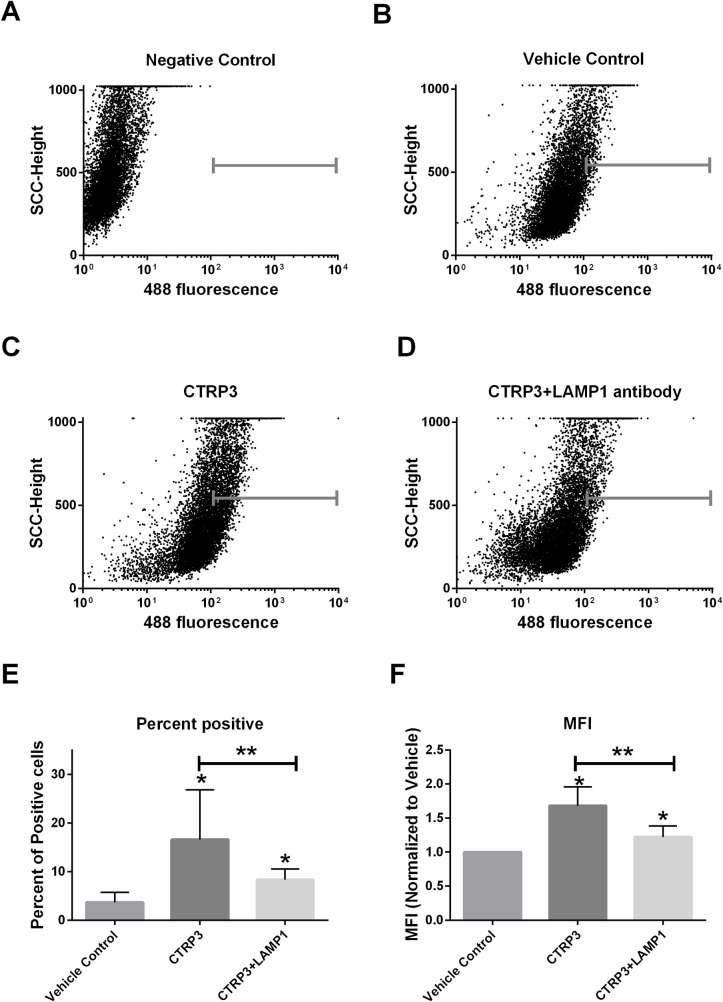
Blocking LAMP1 suppresses CTRP3 binding. H4IIE hepatocytes were grown to confluence in standard media and then treated for overnight ± CTRP3-FLAG (2.5 μg/well) and ± polyclonal LAMP1 antibody (10 μg/well, to block potential CTRP3 binding sites). The cells were then washed and collected in PBS, fixed in 4% formaldehyde, and then incubated with rabbit anti-FLAG antibody followed by fluorochrome-conjugated secondary antibody and analyzed for mean fluorescent intensity (MFI) by flow cytometry. *A-D*, Representative flow data from H4IIE hepatocytes. A MFI >100 was taken as positive and is indicated in the flow plots with a gray bar. *A*, pooled cells with no primary antibody (isotype or negative control). *B*, cell incubated with vehicle, C, recombinant CTRP3-FLAG protein, or *D*, co-incubated with recombinant CTRP3-FLAG protein plus poly-clonal anti-LAMP1. *E*, Percent of the cells that were positive from each experiment. *F*, the MFI ± CTRP3 normalized to vehicle for each independent replicate. Data for *E & F* are from 3 independent replicates performed on different days with separate lots of recombinant CTRP3-FLAG protein with each independent replicate performed in triplicate and reported as mean ± SD. Raw flow cytometry files are attached as supplementary data (S3 File). * p < 0.001 vs. vehicle; ** p < 0.001 vs. CTRP3+LAMP1.

To follow up the LRC-TriCEPS experiment we repeated the FACS experiments with the addition of a polyclonal antibody to LAMP1 in an attempt to disrupt the binding of CTRP3. The co-incubation of H4IIE cells with recombinant CTRP3 and anti-LAMP1 polyclonal antibody was found to significantly attenuate the binding of CTRP3 to H4IIE cells (Fig 5E and 5F).

### CTRP3 Co-IP

**Fig 6:**
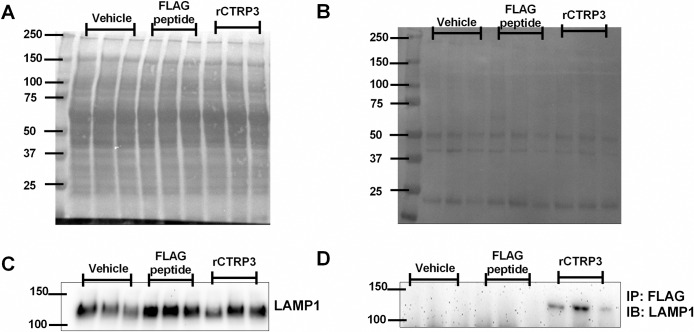
Co-Immunoprecipitation (Co-IP) and Immunoblot Analysis. H4IIE cells were treated overnight with vehicle, FLAG peptide, or recombinant FLAG tagged CTRP3 (rCTRP3). Total protein homogenate (*A & C*) or immunoprecipitate (*B & D*) were separated by SDS-page gel and transferred to nitrocellulose membrane. *A & B*, after immunoblot total protein was visualize by brief incubation with Ponceau S staining solution (5% acetic acid & 0.1% Ponceau S). *C*, LAMP1 in total protein homogenate was similar between treatments. *D*, Co-IP results showing LAMP1 binds to CTRP3. Samples were immunoprecipated with anti-FLAG Affinity Gel followed by immunoblotting with antibodies against LAMP1.

We found that treatment with CTRP3 did not affect LAMP1 protein concentration in total lysate (Fig 6C). To determine whether CTRP3 interacted with LAMP1 protein a Co-IP assay was performed. Immunoblot analysis was able to successfully identify LAMP1 in the CTRP3 Co-precipitate and not in the FLAG peptide or buffer only treated cells, further supporting the hypothesis that CTRP3 and LAMP1 associate *in vivo* (Fig 6D).

## Discussion

### Summary of findings

We successful showed using two different techniques that recombinant purified mammalian-expressed CTRP3 protein binds to the H4IIE hepatoma cells. Further, using real-time oxygen consumption data we demonstrate that pre-conditioning cells with CTRP3 increased oxygen consumption rates. Because electrons produced by free fatty acid (FFA) beta-oxidation enter the electron transport chain at the level of complex 2 while those derived from glucose enter at complex 1, a switch from glucose to FFA utilization is accompanied by higher oxygen consumption rates [45, 46]. Theoretically, the switch from glucose to FFA should cause ~30% increase in OCR [46, 47], if ATP turnover remains unchanged. The ~24% observed increase in oxygen consumption supports previous findings that CTRP3 increases FFA oxidation in hepatocytes [29].

Lastly, the LRC-TriCEPS experiments successfully identified and quantified statistically two proteins as targets for CTRP3: LAMP1 and LIMPII. Additionally, 3 other potential candidates were identified, and although there were not statistically quantified they were reported as putative receptors (Table 1). Follow-up experiments with an anti-LAMP1 polyclonal antibody partially attenuated the binding of CTRP3 to the cells and Co-IP experiments further confirmed that LAMP1 associates with CTRP3.

### Study Limitations

Although we were successfully able to identify to potential proteins which act as the receptor to mediate the effects of CTRP3 there are some study limitations that need to be addressed. This study used an immortalized cell line as a model for hepatocytes. H4IIE cells were chosen because they are a commonly used liver cell culture line that maintains characteristics of intact liver cells and we have shown previously that CTRP3 had a physiological response in these cells (i.e. reduced neutral lipid accumulation [29]). Further, the LRC-TriCEPS protocol requires isolated cells and isolation of hepatocytes from intact liver requires enzymatic dissociation which by definition disrupts cell surface proteins. Future visualization and co-localization studies using intact liver will need to be performed. Additionally, binding and lipid oxidation experiments with primary hepatocytes also needs to be performed. Lastly, the identification of putative receptors that are not exclusively expressed in hepatocytes still leaves open the possibility that the liver-specific *in vivo* effects of CTRP3 [29] are mediated through indirect mechanisms and not necessarily through direct actions of CTRP3 on hepatocytes.

### Hepatic function of CTRP3

Previous work has shown that the novel adipokine CTRP3 has a potent biological effect on the liver [9, 29]. Briefly, both transgenic overexpression of CTRP3 and daily administration of recombinant CTRP3 reduced diet-induced hepatic triglyceride accumulation [29]. Further, acute injections of recombinant CTRP3 significantly lowered hepatic gluconeogenesis upto 8 H post injection [9]. However, the mechanism by which CTRP3 attenuates hepatic triglyceride levels or gluconeogenesis is unknown. Due to similarity of CTRP3 and other members of the CTRP family to adiponectin it has been suggested that the actions of all members of the CTRP family are mediated through the adiponectin receptors: T-cadherin, and adiponectin receptors 1 and 2 [35, 48]. However, our previous work suggests that CTRP3 works through a novel mechanism, as unlike adiponectin, CTRP3 did not increase hepatic AMPK phosphorylation [9, 29, 32]. Moreover, we observed no effect of CTRP3 treatment in skeletal muscle, which has all three of the known receptors for adiponectin [9, 33, 34]. Lastly, we have shown that CTRP3 but not CTRP1 binds to hepatocytes *in vitro* (Fig 2), which supports our hypothesis that CTRP3 is a distinctive member of the C1q/TNF superfamily and functions through a unique receptor.

### Functions of LAMP1 and LIMPII

LAMP1 [Uniprot identifier P14562] is also known as 120 kDa lysosomal membrane glycoprotein (LGP-120) and CD107 antigen-like family member A (CD107a). LAMP1 was initially characterized as a type 1 integral lysosomal membrane glycoprotein that is found in a wide variety of tissues (including the liver) and is commonly used as lysosomal marker [49, 50]. However more recently it has been reported that LAMP1 also shuttles to the plasma membrane, indicating that it could act as a cell surface receptor [51–53]. Further, ~5% of the total LAMP1 protein are located on the plasma membrane due to lysosomal fusion [51, 54]. The transient nature of LAMP1 plasma membrane associate could explain why, in a theoretically homologous cell culture population, only about 15–20% of the cells appeared to be positive for CTRP3 binding (Figs 1 and 2). Regardless, we were able to show that pretreatment with CTRP3 was sufficient to induce a significant increase in oxygen consumption. Although LAMP1, in addition to LAMP2, account for ~50% of lysosomal membrane proteins [49] the exact function for LAMP1 remains elusive, as LAMP1 knockout mice do not appear to have any functional or structural abnormality [49, 52]. Currently, it is believed that LAMP1 is partly responsible for maintaining lysosomal integrity and function [49], and plasma membrane expression of LAMP1 may play a role in tumor cell differentiation and metastasis [49]. The potential role of LAMP1 in mediating hepatic lipid oxidation has not been explored.

Lysosome membrane protein 2 (LIMPII, Uniprot identifier P27615) is also known as Scavenger receptor class B member 2 (SCRB2), 85 kDa lysosomal membrane sialoglycoprotein (LGP85), and as CD36 antigen-like 2. LIMPII was first discovered in a rat liver lysosomal fraction [55] and accounts for ~4% of all lysomal proteins [56]. Although the presence of LIMPII on the plasma membrane of hepatocytes has not been examined, LIMPII has been shown to be essential in the cell-to-cell adhesion of the plasma membrane of cardiac myocytes (intercalated discs) [57]. Although the exact function in metabolism for LIMPII is unknown the closely related protein Scavenger receptor class B member 3 (SCARB3, also known asCD36/FAT) has been implicated in hepatic insulin resistance [58, 59] and immunity [60], both functions implicated with CTRP3. Like SCARB3, LIMPII has been shown to bind to the adhesive glycoprotein thrombospondin-1 [56], which may help mediate cell-to-cell interactions. LIMPII is primarily expressed in the liver, placenta, adrenal cortex and adrenal gland [50] and has already been implicated as an internal receptor responsible for shuttling the enzyme glucocerebrosidase to the lysosome. Glucocerebrosidase metabolizes the sphingolipid glucocerebroside, and when deficient results in Gaucher disease (or the excessive accumulation of the lipid molecule glucocerebroside in cells (i.e. hepatocytes) [61].

Although lacking known cellular signaling functions, both LAMP1 and LIMPII are expressed in hepatic cells, where they are potentially positioned to interact with CTRP3. Further, both of these proteins can be found on the cell surface however, they may act as a co-receptor for an as-yet-unidentified signaling receptor through which CTRP3 transmits metabolic signals.

## Summary and Conclusion

Previous work has shown that the novel adipokine CTRP3 has a potent biological effect on the liver [9, 29]. However, the mechanism by which CTRP3 attenuates hepatic triglyceride levels is unknown. The purpose of this study was to determine if we could successfully use the relatively new methodological approach, LRC-TriCEPS™ method, to identify potential receptors, which mediate the biological effects of CTRP3. We have successfully identified two potential novel receptors using the LRC-TriCEPS™ technique: LAMP1 and LIMPII. Although, the intracellular signaling mechanism remain unknown the identification of the receptors for CTRP3 and other members of this family are an important prerequisite of the development of small molecule drug candidates that work through CTRP3 receptors to exert effects beneficial to human health.

## Supporting Information

S1 Fig Protter visualization of Identified peptides. The identified peptides of LAMP1 are visualized with Protter [62]. Identified peptide sequences are signified by blue circles.
(TIF)

S2 Fig Protter visualization of Identified peptides. The identified peptides of LIMPII are visualized with Protter [62]. Identified peptide sequences are signified by blue circles.
(TIF)

S1 File Raw data files for Fig 2. Unaltered raw flow cytometry files for the data shown in Fig 2.
(ZIP)

S2 File Raw data files for Fig 4. Unaltered raw flow cytometry files and excel spreadsheet of Mass spectrometry output for the data shown in Fig 4.
(ZIP)

S3 File Raw data files for Fig 5. Unaltered raw flow cytometry files for the data shown in Fig 5.
(ZIP)
