# Supplementary figures and images for: Identification of Putative Receptors for the Novel Adipokine CTRP3 Using Ligand-Receptor Capture Technology
Source: PLoS One. 2016 Oct 11;11(10):e0164593. doi: 10.1371/journal.pone.0164593 (PMC5058508; doi:10.1371/journal.pone.0164593)

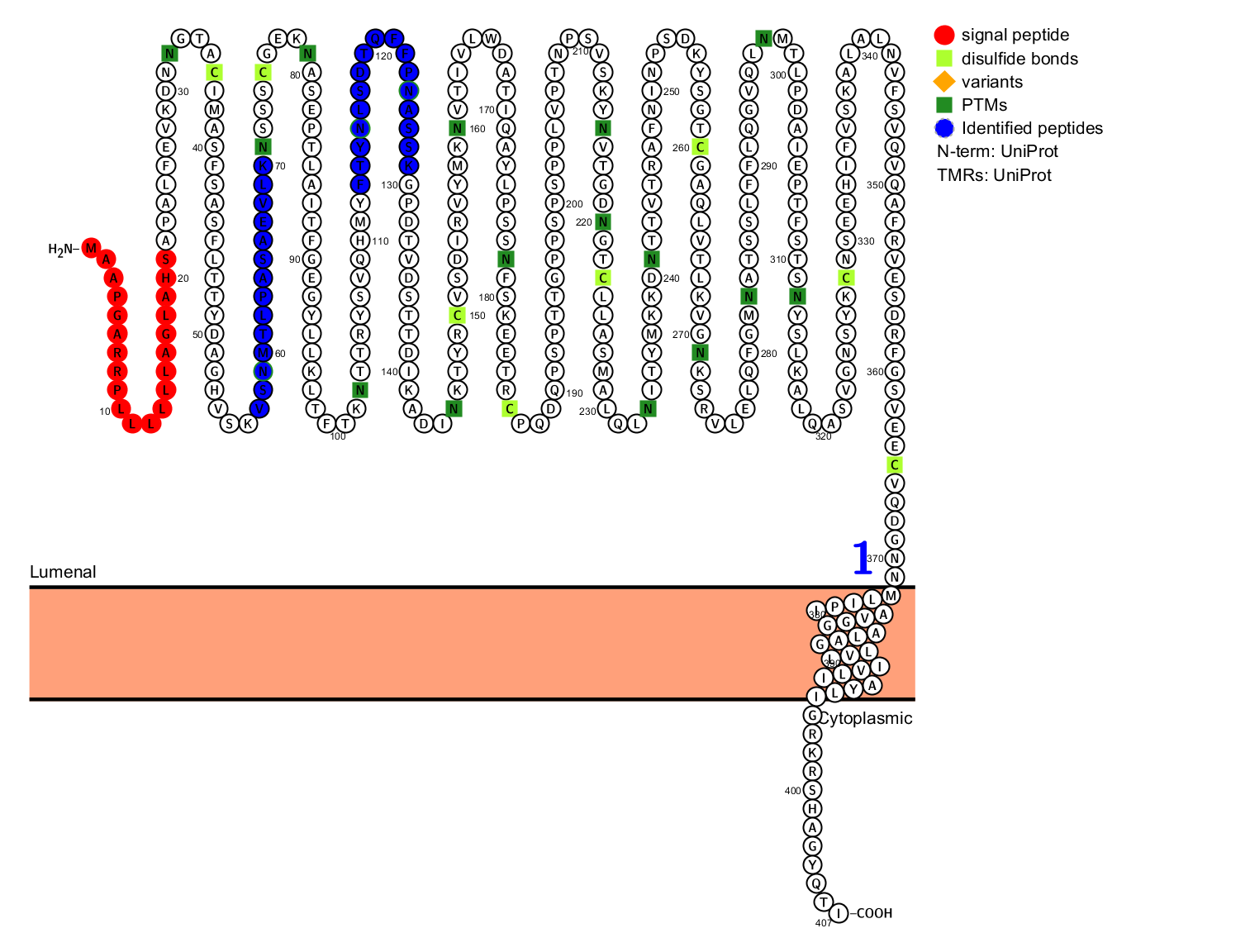

Supplement: S1 Fig — The identified peptides of LAMP1 are visualized with Protter [62]. Identified peptide sequences are signified by blue circles. (TIF) [file pone.0164593.s001.tif]

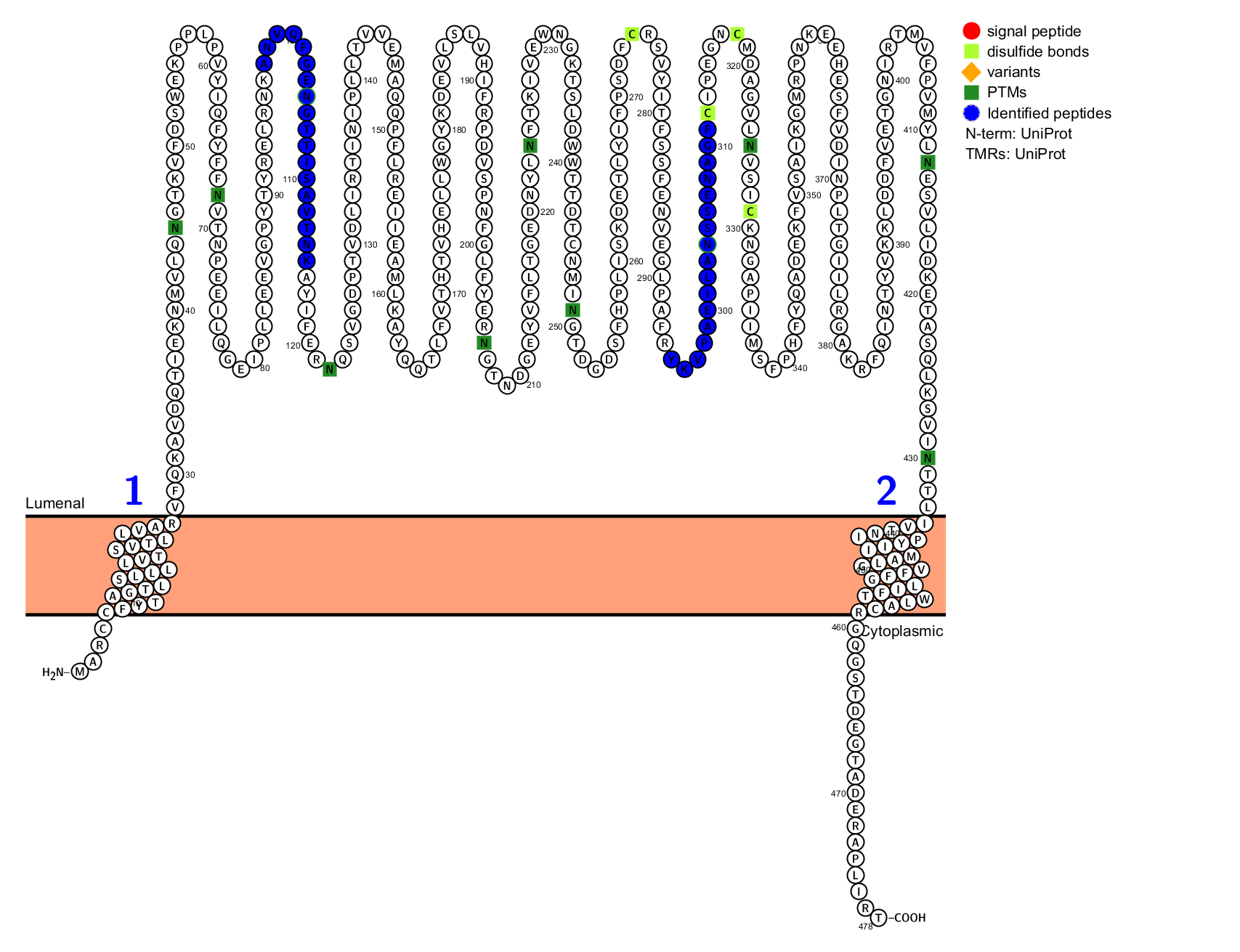

Supplement: S2 Fig — The identified peptides of LIMPII are visualized with Protter [62]. Identified peptide sequences are signified by blue circles. (TIF) [file pone.0164593.s002.tif]
